# Ice-sheet-driven methane storage and release in the Arctic

**DOI:** 10.1038/ncomms10314

**Published:** 2016-01-07

**Authors:** Alexey Portnov, Sunil Vadakkepuliyambatta, Jürgen Mienert, Alun Hubbard

**Affiliations:** 1 CAGE—Centre for Arctic Gas Hydrate, Environment and Climate, Department of Geology, UiT The Arctic University of Norway, 9037 Tromsø, Norway

## Abstract

It is established that late-twentieth and twenty-first century ocean warming has forced dissociation of gas hydrates with concomitant seabed methane release. However, recent dating of methane expulsion sites suggests that gas release has been ongoing over many millennia. Here we synthesize observations of ∼1,900 fluid escape features—pockmarks and active gas flares—across a previously glaciated Arctic margin with ice-sheet thermomechanical and gas hydrate stability zone modelling. Our results indicate that even under conservative estimates of ice thickness with temperate subglacial conditions, a 500-m thick gas hydrate stability zone—which could serve as a methane sink—existed beneath the ice sheet. Moreover, we reveal that in water depths 150–520 m methane release also persisted through a 20-km-wide window between the subsea and subglacial gas hydrate stability zone. This window expanded in response to post-glacial climate warming and deglaciation thereby opening the Arctic shelf for methane release.

## Abstract (web summary)

Methane release across the Arctic continental shelf has been attributed to modern dissociation of gas hydrate, accelerated by ocean warming. Here, the authors show that thermogenic methane was stored as subglacial gas hydrate during the last glaciation, and subsequently released following ice sheet retreat.

## Graphical abstract

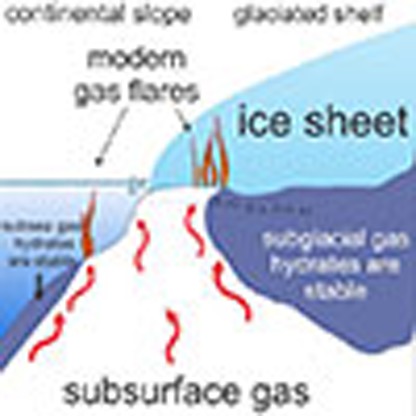

Natural gas can exist in solid form of crystalline ice-like structures known as gas hydrates that are stable within the subsurface under high-pressure and low-temperature conditions bounded by the gas hydrate stability zone (GHSZ). The kinetics of hydrate formation and dissociation also critically depends on the supply and composition of gas and liquid water within available pore space of sediments, hence even under an appropriate envelope of GHSZ pressure and temperature conditions, gas hydrates are not, *per se*, guaranteed^1^. However, wherever persistent subsurface methane (or heavier fractions of natural gas) and water coexist within available pore space, then the GHSZ is a robust indication of the conditions under which gas hydrate is likely to form. The present distribution and stability of gas hydrates beneath oceans and permafrost, along with their potential to release large fluxes of methane and other potent greenhouse gases, are fundamental to determining long-term atmospheric composition and its impact on climate change. Previous research reveals that subglacial soils, lakes, peatlands and marine sediments can store significant reserves of carbon within a GHSZ beneath the palaeo-ice sheets that covered North America and also beneath the Antarctic ice sheet today^2^,^3^. It has been argued that as a result of active methanogenesis, this significant carbon pool could provide a major contribution to global atmospheric methane emissions and composition following deglaciation of Antarctica^4^. However, to date, few studies have investigated how gas hydrates responded to past climate change—specifically—the impact of extensive ice-sheet expansion on gas hydrate stability and dissociation during the last glaciation. In particular, three leading research questions warrant attention: how did the subglacial footprint of the former ice-sheet affect the GHSZ? How could this GHSZ govern methane storage and release across the glaciated margin? How could post-glacial ice-sheet retreat impact on this former subglacial gas hydrate reservoir?

Persistent and extensive discharge of methane gas flares into the water column offshore of Prins Karls Forland (PKF), western Svalbard has been reported since they were first observed in 2008 (refs 5, 6). More than 1,000 individual gas flares, predominantly ejecting methane (C_1_ ∼98.9–99.9%) from known hydrocarbon sources^7^ cluster across a broad zone of the seabed between 80 and 420 m depth^8^ (Fig. 1). Gas flares can be grouped in two distinct sets based on depth: a deep zone that spans 380–420 m below sea level (m.b.s.l.) and a shallow zone between 80 and 130 m.b.s.l. Deeper gas flares can theoretically be associated with the base of the present-day GHSZ, which pinches out on the seabed at 396 m water depth^6^. It has been argued that a temperature increase of 1 °C within the West Spitsbergen Current bottom water and/or annual fluctuations of 0.6–4.9 °C has been sufficient to force a downslope migration of the GHSZ in this area from 360 to 396 m.b.s.l. over the past three decades^5^,^6^. Despite a lack of records of vertical fluid flow in the study area, U/Th isotope analyses on authigenic carbonate records reveal that there has been significant methane flow-induced precipitation at the deeper gas flares sites since at least 3 ka (ref. 5). In contrast, shallow gas flares cluster across the main ridge of the Forlandet moraine complex^9^ (Fig. 1). The region is characterized by a sediment blanket that diminishes from several hundred metres over the continental slope to a minimum of a few tens of metres on the shelf^10^,^11^. The tectonically induced Forlandsundet graben (Fig. 1) is infilled with several kilometres thick sediment section^12^,^13^, and hence has major potential to host free gas. Sediment thickness to the southeast at Isfjorden varies up to ∼100 m (ref. 14). Hydrocarbon source rocks are extensive in the Svalbard region, including Triassic and Early Jurassic formations^7^, as well as organic-rich Miocene deposits^15^. The major hydrocarbon reservoirs within our study area are concentrated within Early and Middle Triassic source sequences^7^. Natural gas including thermogenic methane—the lightest hydrocarbon fraction—is generated under high pressure and temperatures up to 200 °C at depth within sedimentary basins from a mixture of insoluble organic compounds known as kerogen^16^. In 1992, during exploratory drilling in Svalbard, gas blow outs from depths of 630 m beneath today's sediment surface brought operations to a complete halt^17^.

It is well established that the ultimate stable ice-sheet stand across the West Spitsbergen shelf was concurrent with maximum thickness and horizontal extent (representing the Last Glacial Maximum (LGM) stage) and persisted for at least 5 kyr (20–15 ka)^18^,^19^. Onshore and offshore radiocarbon dating^20^, cosmogenic ^10^Be surface exposure dating^20^,^21^, numerical modelling^22^, offshore high-resolution multibeam^23^ and seismic surveys^9^ reveal a LGM sequence that extended across the continental margin to the shelf break. By inference, the ice sheet in this sector was cold based and covered the Spitsbergen continental shelf to a distance of ∼45 km offshore (∼150 m.b.s.l.; Fig. 1). Field evidence further reveals that the ice surface was at least ≥473 m above the present sea level (m.a.s.l.) as determined from the ^10^Be exposure age of the boulders over the PKF, and ∼700–900 m.a.s.l. over the west Spitsbergen margin^21^,^22^ (Fig. 1). Wet-based, fast-flowing ice streams discharged across the shelf from Kongsfjorden and Isfjorden, and bounded the cold-based ice lobe that flowed across our study area and west of PKF^24^.

Radiocarbon (^14^C) dating of whale bones and mollusk shells sampled on the raised beaches define the earliest post-LGM marine limits on the northern PKF (25 m.a.s.l.) and western Spitsbergen (45–48 m.a.s.l.)^25^,^26^ (Fig. 1). The resulting sea level curves define a local glacio-isostatic loading scenario that was at least 100 m at PKF and western Spitsbergen. The time scale for ice-sheet stabilization at its LGM stand and subsequent retreat was 20–15 ka in this region of western Svalbard, followed by complete deglaciation of the continental margin that was complete by 12–10 ka (ref. 24). This glacial chronology is consistent with an initial phase of relatively slow post-glacial isostatic rebound of 1.5–5 m ka^−1^ for West and North Svalbard that commenced at ∼13–12 ka, followed by an episode of accelerated uplift (15–30 m ka^−1^) between 10.5 and 9 ka (ref. 27).

In this study, we model the impact of the paleo-ice sheet on the GHSZ offshore of western Svalbard by integrating geophysical mapping with the glacial geology. Our results reveal a potentially large subglacial gas hydrate reservoir that accumulated under high-pressure/low-temperature LGM conditions and that would have subsequently dissociated, releasing methane during deglaciation and marine incursion.

## Results

### Modelling predicts thick GHSZ for 20 ka

**Figure 1:**
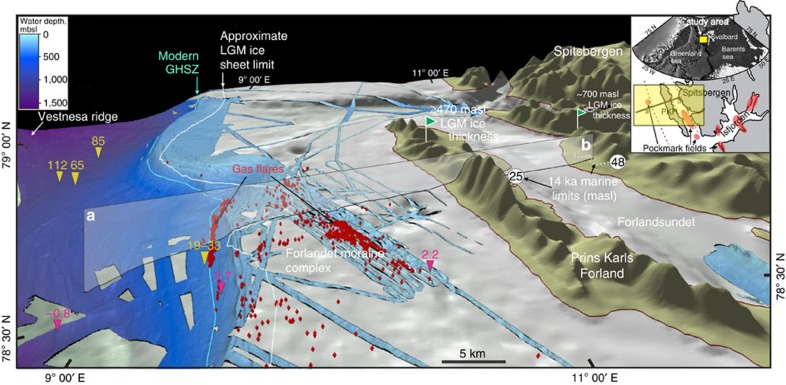
The West Svalbard shelf at present. The western Svalbard margin (IBCAO v.3 (ref. 51) in grey and high-resolution multibeam data in the blue scale—see Methods) showing the observational compilation used in the modelling experiments along the transect a to b (semi-transparent vertical curtain). Yellow and red triangles show geothermal temperature gradients (19–112 °C km^−1^)^5^,^35^,^36^ and average long-term bottom water temperatures (−0.8 to 2.2 °C)^32^, respectively, which were used to constrain the LGM boundary conditions along the transect. Also indicated are the LGM marine limits^25^,^28^, used to estimate isostatic loading (25 and 48 m in white rounds), minimum ice-surface elevation^21^ (green flags), location of modern GHSZ, approximate LGM ice-sheet limit and modern gas flare locations (see Methods). Inset is the location of the study area in respect to pockmark fields (red ovals) and major tectonic lineaments of Hornsund fracture zone (dashed brown lines) across the western Svalbard margin.

**Figure 2:**
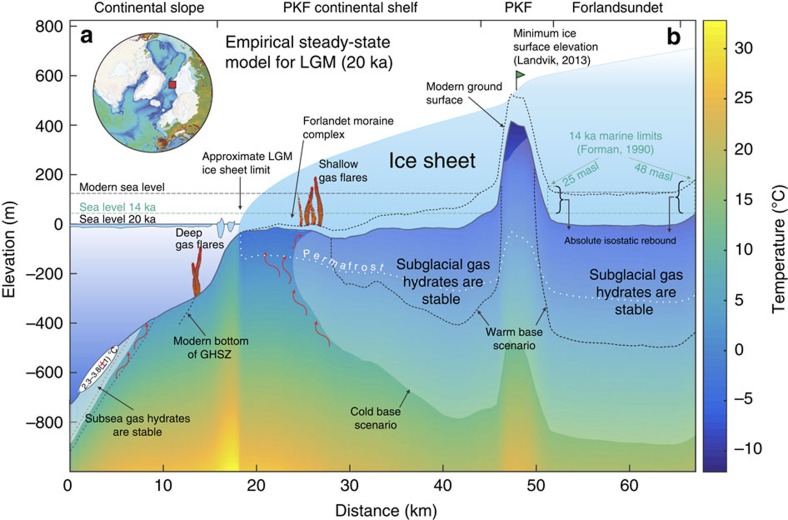
Empirical steady-state model for the Last Glacial Maximum. Modelled for LGM (20 ka) ice-sheet and gas hydrate reconstruction showing both subsea and subglacial GHSZ in a minimum cold-based and warm-based ice-sheet scenario. The elevation datum is set at the LGM sea level (−120 m compared with present). Black dashed line shows the base of GHSZ in a warm-based ice-sheet scenario (pressure melting point temperature along the ice-sheet bed surface). Red and blue dashed lines show the shift of subsea GHSZ under ±1 °C bottom water temperature deviation. Gas-hydrate-free window existed between subsea and subglacial GHSZ during the LGM within the upper margin, leaving the potential for methane release (red arrows). Present-day seabed gas flares are confined to modern and ancient subsea and subglacial GHSZ pinch-out areas.

The GHSZ model applied here uses a reconstructed LGM ice-sheet configuration, constrained by the available empirical evidence as described above^9^,^23^,^28^. Field data provide minimum constraints on ice-sheet thickness, isostatic loading and offshore extent to provide boundary conditions for two-dimensional, steady-state ice-sheet modelling under a perfect-plastic ice-flow assumption^29^,^30^ (Figs 1 and 2; see Methods). This LGM, cold-based ice-sheet reconstruction attained ∼700-m thickness over Spitsbergen and thinned westward where it terminated into the ocean. We set the sea level datum to conform to the global eustatic sea level curve^31^, with zero-elevation aligned to its 20 ka level (120 m below the present; Fig. 2). Observations indicate significant subsidence of subglacial ground surface, accruing eastward and attaining ∼128 m under the West Spitsbergen margin (see Methods). LGM bottom water temperatures range from +2.3 to +3.8 °C from West to East along the transect (Fig. 2; Supplementary Fig. 1). These temperatures are ∼1 °C higher than the present-day average bottom water temperatures^32^ due to episodes of intensive Atlantic Water inflow and cold freshwater discharge from glaciated margins^33^,^34^. The juncture of the ocean–ice sheet interface critically controls the ground surface temperature change between the submarine and subglacial environments. It abruptly decreases from +3.5 °C within the ocean to −4.5 °C within the subglacial environment and consequently impacts significantly on the temperature distribution in the lower subsurface (Fig. 2; Supplementary Fig. 1).

These LGM conditions, combined with present-day heat-flow measurements^5^,^35^,^36^, were coupled with a thermal diffusion model to yield the two-dimensional distribution of subsurface temperature during the LGM. This is subsequently used to model an extensive subsea and subglacial GHSZ with a thickness in excess of 800 m below Forlandsundet that declines westward to where it abruptly tapers-out under the Forlandet moraine complex (Fig. 2). In this configuration, the subglacial GHSZ system resembles its deep offshore analogue, but with the ice sheet providing the high-pressure- and low-temperature loading conditions. Approximately, 20 km westward from where the subglacial GHSZ terminates, the subsea GHSZ pinches out across the seabed at ∼400 m water depth (∼520 m below the present sea level; Fig. 2). Here and in contrast to the subglacial situation, the subsea GHSZ attained a maximum thickness of ∼160 m beneath the western and deepest submerged part of the transect (Fig. 2).

Modelling also reveals an upper margin window for potential methane release since the subsea and subglacial GHSZ configurations leave the ice-sheet margin with a GHSZ-free region. Hence, continuous gas release was possible, indeed, likely across the upper continental slope during the LGM, and, potentially, throughout the last glacial cycle. A cold-based ice-sheet scenario that also agrees with available field data predicts at least 100 m of thick subglacial permafrost above a 0 °C isotherm, abruptly terminating under the shelf break due to the thermal diffusion beneath the subglacial/subsea interface (Fig. 2).

### GHSZ is sensitive to environmental changes

The GHSZ modelling presented here is bounded by the subglacial and subsea temperature distributions associated with an optimal LGM ice-sheet reconstruction constrained by the glacial geological record and cosmogenic dating. We investigate the sensitivity of the modelled GHSZ to alteration of these boundary conditions by, first, changing the subglacial thermal conditions from a cold to a warm-based scenario and, second, by increasing the ice-sheet reconstruction from a minimum thickness in accordance to available field data, to a maximum profile.

The transition from a cold to temperate subglacial conditions (defined as being at pressure melting point) leads to a twofold decrease in GHSZ thickness (Fig. 2; Supplementary Fig. 1). Moreover, its surface exposure point shifts eastward ∼3 km across the modern Forlandet moraine complex. Model sensitivity to oceanic thermal conditions was investigated through a ±1 °C deviation of the bottom water temperature profile and reveals a reciprocal ∼±1.5 km shift of the subsea GHSZ surface exposure point along the continental slope, fixing it between 390 and 450 m below LGM sea level (Fig. 2). The subsurface geothermal heat-flow pattern is interpolated from a sparse array of borehole measurements from across the region (see Methods) and also represents a source of uncertainty. The sensitivity experiments do though confirm that the approach we adopt along with our results are robust and indicate that even an increase in lithospheric temperature gradients by 10 °C km^−1^ shifts the base of GHSZ upwards by some 100 m at most and hence, does not substantively detract from our findings.

The ice-sheet surface profile that optimally concurs with the available field evidence is achieved with a yield strength for perfect-plastic ice-sheet flow of 40 kPa (ref. 29). An ice-sheet reconstruction with such a low-yield strength is glaciologically valid^30^ and within bounds of published values under temperate subglacial conditions provides conservative, end-member, boundary conditions for pressure and thermal loading of the subglacial GHSZ during the LGM (that is, it yields the thinnest ice-sheet profile with warmest basal temperatures). Yield stress, if increased to a more typical value of 80 kPa for a cold-based ice sheet^29^,^30^ yields an increase both in ice thickness to a maximum of 950 m and associated subglacial GHSZ to ∼900 m beneath Forlandsundet (Supplementary Fig. 1). Under this maximum ice thickness reconstruction, the surface exposure point of subglacial GHSZ shifts ∼2.5 km westward, reflecting the steeper ice profile to the margin.

## Discussion

Post-LGM eustatic sea level rise shifted the subsea GHSZ ∼6.5 km laterally up the continental slope, where it attained its present location at 396 m.b.s.l. and is, today, marked by abundant gas flares that align parallel to the slope (Figs 1 and 2). We hypothesize that the lateral displacement of subsea GHSZ developed in parallel with the migration of gas-saturated fluids along the base of GHSZ (Fig. 2). This would impact on the formation of multiple fluid flow features within the glacio-marine sedimentary sequence as observed below the modern subsea GHSZ in recent high-resolution seismic data^11^,^37^.

**Figure 3:**
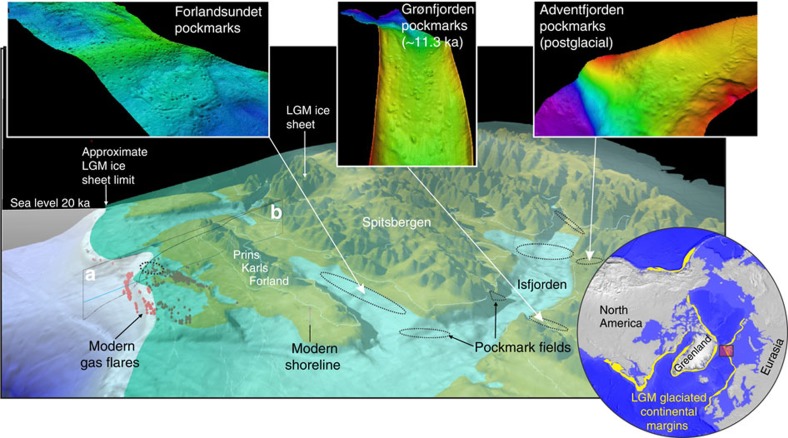
Conceptual reconstruction of the glaciated western Svalbard margin. Dotted ellipses reveal the location of discovered pockmark fields that we infer to be due to the post-LGM retreat of the ice sheet. Upper insets demonstrate pockmark fields in high-resolution multibeam data. Lower inset schematically shows the widespread distribution of the formerly glaciated margins at the LGM across the Arctic, for which the western Svalbard margin is a potential GHSZ analogue.

We collate over 1,000 pockmarks and active gas flares across the shallow West Spitsbergen shelf and fjord environments (Fig. 3)^14^,^23^,^38^. Gas from deeper hydrocarbon sources is likely to migrate along the tectonic lineaments. Alternatively, release from thawing relic permafrost also should be considered^8^. However, 546 of 1,304 individual pockmarks studied in Isfjorden are confined to thrust faults, indicating focused fluid flow migration^38^. The present-day gas discharge across the shelf west of PKF is coincident with the exposure point of subglacial GHSZ modelled here (Figs 1 and 2). This apparent match between our modelled GHSZ using a range of LGM ice-sheet scenarios and the incidence of observed gas flares is a robust yet paradoxical coincidence, since the subglacial GHSZ might have been expected to follow the post-LGM pattern of deglaciation. However, at present, there is no convincing observational evidence for pockmark and related gas flare activity that synchronously shifted eastward with on-going ice retreat and deglaciation. During its LGM stand, the West Spitsbergen ice sheet provided continuous high-pressure and thermal subglacial conditions for a period of at least 5 kyr (ref. 18) —sufficient time for the establishment of an extensive and persistent GHSZ that hence had the potential for the formation of gas hydrates in regions with favourable geological setting^39^. On deglaciation, the former subglacial environment associated with the ice sheet was subject to marine incursion and became exposed to shallow marine environments, accompanied by decreased hydrostatic pressure and increased bottom temperatures related to the inflow of ∼2–3 °C warm Arctic water. This major environmental change was on-going and took place within 7 kyr (refs 19, 24) and resulted in a transient reduction of the previous thick subglacial GHSZ and associated permafrost layer. As a result, this sequence will have enabled triggering of seabed release of methane formerly trapped beneath the ice sheet in the form of gas hydrate, fuelled by deep hydrocarbon reservoirs that continued to drive methane upward through to the present day^40^,^41^. Active fluid flow and pockmark footprints across the vast area of western Spitsbergen margin have been mapped outside and inside the fjords (Fig. 3), coincident with known deeper petroleum reservoirs^7^. Our post-glacial scenario for natural greenhouse gas release is supported by age constrains for Isfjorden pockmarks, which have been dated as post-glacial, and agree with the age estimation for Grønfjorden (southernmost branch of Isfjorden) pockmarks of ∼11.3 ka (ref. 14) (Fig. 3). Such a geological setting appears to be characteristic for extensive post-LGM seabed gas escape across the Arctic margin and potentially across the US east coast continental margins where thousands of gas flares have been observed^42^.

High pressures to at least 50 MPa coupled with temperatures −5 °C or cooler under the LGM ice sheet likely promoted expansion of the gas hydrate reservoir beneath the western Svalbard margin, but also left a ∼20-km-wide gas-hydrate-free zone, which served as a corridor for upward migration of fluids and release of methane throughout the LGM. The North West Svalbard shelf and upper slope provides a robust test bed for understanding gas hydrate formation and dissociation across a formerly glaciated continental margin and reference point for further natural greenhouse gas release studies in Polar Regions. Former continental margins with a similar paleo-subglacial footprint and legacy extend some 20,000 km in the northern hemisphere where major ice sheets advanced across western Eurasia shallow shelves, Greenland and eastern and western American shelves (Fig. 3, inset). We infer that the ongoing deglaciation of these continental margins between 18 and 12 ka (ref. 43), many of which are underlain by deep hydrocarbon reservoirs, would have likely been accompanied by widespread dissociation of gas hydrates and associated increase of methane flux from the seabed. In the outer continental shelves where the eustatic signal outpaced isostatic rebound, methane emissions from recently inundated shallow shelves (first tens of metres) would have been expelled into the atmosphere, similar to present-day process of methane transport across the shallow East Siberian Arctic Shelf^44^. This study not only implies the potential for significant gas hydrate storage and release capacity during past glacial/inter-glacial conditions but is also significant in its implication for current and future greenhouse gas release under the ongoing thinning and retreat of contemporary ice sheets and glaciers^45^.

## Methods

### Ice-sheet configuration and bed surface temperature modelling

The equilibrium profile of the West Svalbard ice sheet was obtained using a steady-state model based on the refined perfect-plastic assumption for ice flow^29^,^30^,^46^. Under these conditions, plastic-flow yielding a steady-state ice-sheet thickness distribution (H) occurs when basal shear stress (*τ*_b_) is equal to the yield stress (*τ*_0_) at all points across the bed:


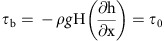


where *ρ* is the density of ice, *g* is the gravitation constant and dh/dx is the ice-surface slope. This equation can be rearranged and applied to reconstruct former ice sheets using observational constraints that indicate its maximum extent, such as offshore moraines sequences, and its former surface from, for example, cosmogenically dated erratics and trimlines. To reconstruct the ice-sheet surface, the above equation is integrated numerically^20^, from the margin (H=0) under a given subglacial topographic profile with an appropriate value of yield strength (in our case 40 and 80 kPa), which for grounded ice masses can fall between 40 and 100 kPa (refs 30, 47).

Reconstruction of the LGM isostatic loading involved adjustment of past marine limits to account for relative sea level rise. Oldest post-LGM marine limits discovered on the PKF (25 m.a.s.l.) and Spitsbergen margin (48 m.a.s.l.) were dated as 14 ka (refs 25, 28; Fig. 2). Thus, given the ∼80 m sea level rise since 14 ka, we estimate the absolute isostatic rebound as ∼105 and ∼128 m for PKF and Spitsbergen margin, respectively (Fig. 2).

We determine the steady-state cold-subglacial temperature distribution based on conservation of energy at the bed from vertical diffusion, advection and frictional heating as implemented and validated for various glaciers and the Antarctic ice sheet^30^,^48^,^49^. As implemented at Taylor Glacier, Dry valleys^50^, for its application to the LGM ice sheet in western Svalbard (Supplementary Fig. 1), we use a lapse rate of −0.007 °C m^−1^, an accumulation rate of 0.3 m a^−1^ and mean annual temperature at the margin of −14 °C. For the warm-bed situation, basal temperatures are assumed to be at pressure meting point calculated according to H × 8.70 × 10^−4^ (ref. 30).

### Subsurface heat-flow model

We sampled the seafloor depths along the transect (Fig. 1) at every 100 m from IBCAO v.3 gridded bathymetric data^51^ and adjusted the depths to sea level at 20 ka (ref. 31). Then, a 671 × 1,000 cell temperature grid (100 × 1-m cell dimensions) was generated, with its upper boundary at the seafloor and basal boundary 1 km below seafloor. To define initial temperature conditions, existing heat-flow measurements^5^,^35^,^36^ (Fig. 1) located near the selected transect were utilized. A spin-up model temperature grid was generated by assuming a one-dimensional linear temperature gradient, with ocean bottom water temperatures^32^ adjusted to 20 ka (refs 33, 34) and ice-bottom temperatures (where ice exists) as the top boundary condition (Supplementary Fig. 1). To constrain the thermal diffusivity, we assumed a constant thermal conductivity of 2 W m^−1^ K^−1^, average density of 1,900 kg m^−3^ for the bulk sediments and an average specific heat capacity of 2,000 J kg^−1^ K^−15^.

Assuming no significant *in situ* generation of heat, we run the two-dimensional finite difference heat-flow model^52^ for 5 kyr (assuming no significant variation of ice extent, sea level and ocean bottom temperatures in the study area during this period). The model is run for two different ice-bottom temperatures (Supplementary Fig. 1) and two different ice-sheet thicknesses, corresponding to 40 and 80 kPa yield stress.

### Gas hydrate stability modelling

To identify the base of methane hydrate stability, we integrate results from our heat-flow model with theoretical hydrate stability phase diagrams generated using the CSMHYD program^1^, which uses an algorithm based on Gibbs energy minimization and account for different pressure and temperature conditions, the composition of gas forming hydrates and the presence of inhibitors of hydrate formation (for example, salt). Assuming pure methane gas and a constant pore water salinity of 35‰, CSMHYD program estimates the pressure at which hydrates are stable for any given temperature. With temperature constrained from our heat-flow model, we estimated the hydrostatic pressure at each cell location using standard values for density of seawater (1,027 kg m^−3^) and acceleration due to gravity (9.8 m s^−2^). This pressure grid is then compared with theoretical predictions from CSMHYD program to determine the hydrate stability at each cell location.

### Hydroacoustic data

High-resolution bathymetry data have been acquired in 2004–2014 onboard RV ‘Helmer Hanssen' by the Arctic University of Norway (UiT) using Kongsberg-Simrad EM300 multibeam echosounder system. Multibeam data were gridded with the cell 7–10 m in the water depths ≤200 and 15–20 m in the water depths >200 m, which provided sufficient resolution for pockmark detections. Locations of gas flares offshore PKF were derived from previously published study^8^. New water column data, acquired in 2014 with Simrad ER-60 echosounder during UiT cruise onboard RV ‘Helmer Hanssen' has been processed using Fledermaus software and included in the current study.

## Additional information

**How to cite this article:** Portnov, A. *et al.* Ice-sheet-driven methane storage and release in the Arctic. *Nat. Commun.* 7:10314 doi: 10.1038/ncomms10314 (2016).

## Supplementary Material

Supplementary Information Supplementary Figure 1
