# Supplementary material for: Ice-sheet-driven methane storage and release in the Arctic
Source: Nat Commun. 2016 Jan 7;7:10314. doi: 10.1038/ncomms10314 (PMC4729839; doi:10.1038/ncomms10314)
Supplement: Supplementary Information — Supplementary Figure 1 [file ncomms10314-s1.pdf]

Supplementary Figure 1. Sensitivity of subglacial gas hydrate stability zone.

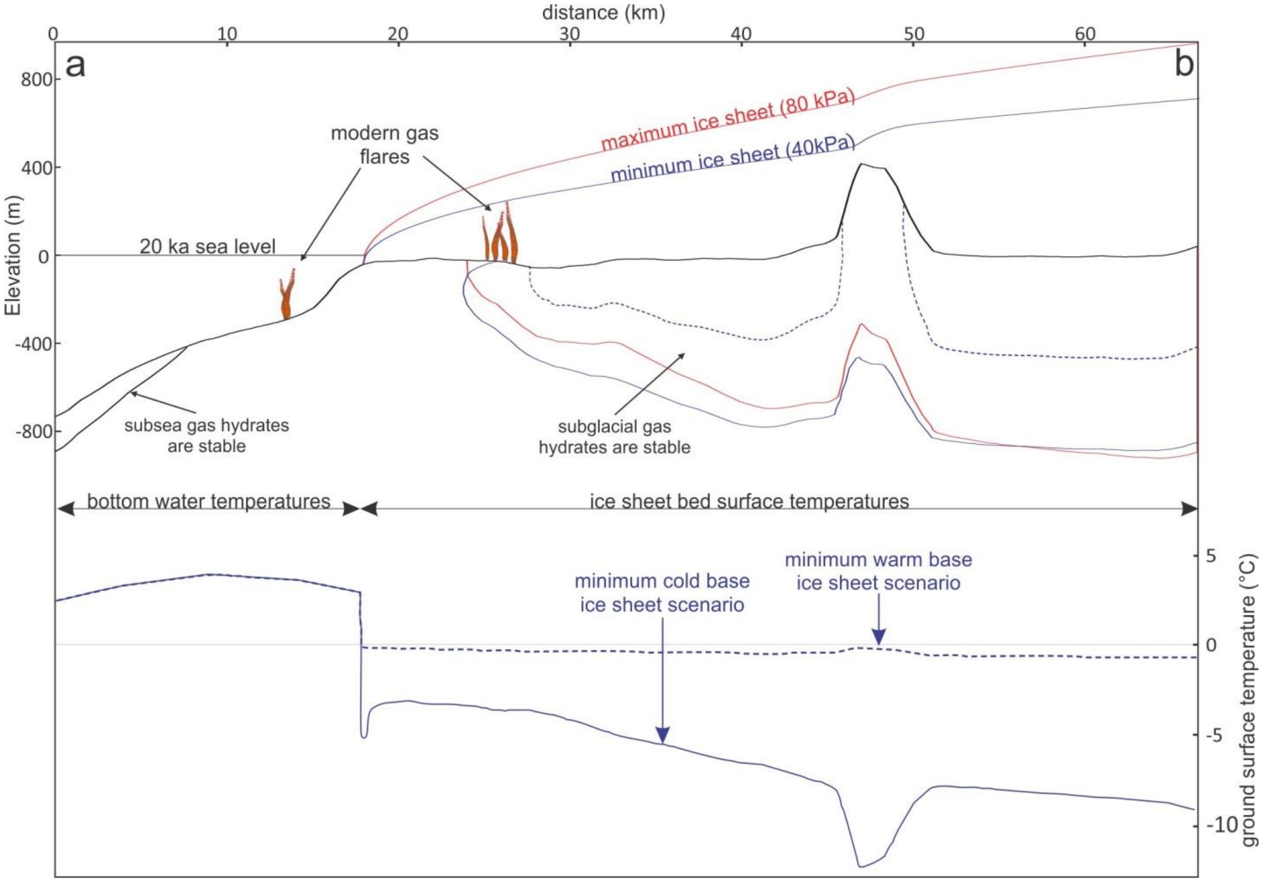

Supplementary Figure 1 shows sensitivity of subglacial GHSZ modeling depending on the configuration of the ice sheet and bed surface temperatures. The least GHSZ thickness (blue dashed line in the upper section) is achieved under the minimum (empirical) warm-based ice sheet. Cold base scenarios for minimum and maximum ice sheet are shown with solid blue and red lines respectively. Blue curves in the lower section show the distribution of ground surface temperature along the transect under the minimum cold-based (solid) and warm-based (dashed) ice sheet.
